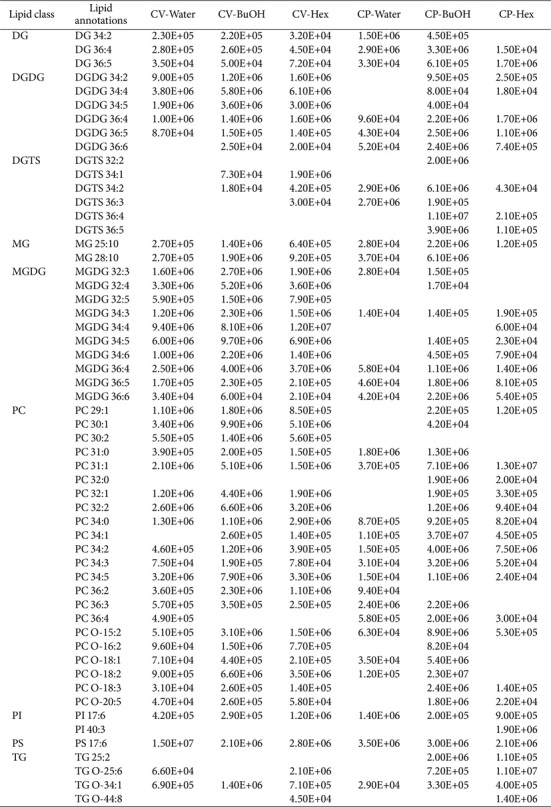# Chemical Profiling and Immune-Stimulating Activity of Solvent Fractions Derived from Dietary *Chlorella*

**DOI:** 10.4014/jmb.2503.03021

**Published:** 2025-06-12

**Authors:** Seung-Woo Jo, Priyanka Velankanni, Nhung Do Thi Cam, Chau Hoang Bao Nguyen, Jin-Young Jeon, Bo-Ra Kim, Dae-Hee Lee, Choong-Gu Lee, Jin-Soo Park

**Affiliations:** 1 Center for Natural Product Systems Biology, Korea Institute of Science and Technology (KIST), Gangneung 25451, Republic of Korea; 2 Department of Marine Bio Food Science, Gangneung-Wonju National University, Gangneung 25457, Republic of Korea; 3 Natural Product Applied Science, KIST School, University of Science and Technology, Gangneung 25451, Republic of Korea; 4 BIO R&D Center, Ingredient Business Unit, Daesang Corporation, Seoul 07789, Republic of Korea

**Keywords:** *Chlorella vulgaris*, *Chlorella protothecoides*, immune-stimulating activity, lipid, 5’-methylthioadenosine (MTA)

## Abstract

*Chlorella*, a genus of single-celled green algae, is widely industrially cultivated as a dietary supplement ingredient due to its high protein content and various nutrients. In this study, the immune-stimulating effect of solvent fractions prepared from *Chlorella vulgaris* (CV) and *Chlorella protothecoides* (CP) was evaluated through the expression of immune-related factors, such as TNF-α and IFN-γ in T cells obtained from mice. Both *Chlorella* species showed immunostimulatory effects in hexane and butanol fractions, but CV increased TNF-α only, while CP elevated both TNF-α and IFN-γ. In addition, the lipid profile of *Chlorella* was characterized using non-selective LC-MS/MS-based metabolomic analysis. According to the lipidomic results, the two species differed in lipid composition, with PC 32:1, PC 32:2, PC 34:5, DGDG 34:4, and DGDG 34:5 being enriched in the active fractions of CV, and PC 34:1, PC 34:2, PC O-15:2 and DGTS 34:4 being abundant in the fractions of CP. Furthermore, 5’-methylthioadenosine (MTA), which was detected in both *Chlorella* species, also exhibited immunostimulatory properties.

## Introduction

There is a growing interest in sustainable and environmentally friendly food resources, driven by the scarcity of natural resources and the agricultural challenges posed by rapid environmental changes [1, 2]. Sustainable food sources are also expected to not only provide essential nutrition, but also contribute to improved human health. This is made possible through the bioactive properties of these foods and ingredients, which have been extensively studied and enhanced through advancements in natural product research and biotechnology. Among food components, saturated fats adversely affect vascular function, whereas unsaturated fats have been reported to lower blood pressure, improve arterial compliance in patients with type 2 diabetes and dyslipidemia, and reduce the risk of coronary heart disease [3-5]. Unsaturated fats, which are effective in improving cardiovascular disease, affect the effector and regulatory functions of innate and adaptive immune cells. Imbalances in saturated/unsaturated fatty acids affect the homeostasis of the immune system and contribute to the development of many allergic, autoimmune, and metabolic diseases [6, 7].

CD4+ and CD8+ T cells are essential components of the adaptive immune system, each playing unique roles in managing immune responses to infections, tumors, and other challenges [8]. CD4+ T cells, often called helper T cells, play a crucial role in coordinating adaptive immune responses by cytokine secretion. These cytokines regulate the activation and functions of other immune cells, including macrophages, B cells, and CD8+ T cells [9]. CD8+ T cells, also known as cytotoxic T lymphocytes (CTLs), are primarily involved in targeting and destroying infected or abnormal cells. They achieve this also by producing significant levels of IFN-γ and TNF-α, which can induce cell death in their targets [10]. Assessing the production of TNF-α and IFN-γ by CD4+ and CD8+ T cells in response to specific stimuli is critical for understanding their immune stimulatory effects. TNF-α often works in synergy with IFN-γ to enhance immune responses. Together, the production of these cytokines by CD4+ and CD8+ T cells plays a pivotal role in shaping immune responses, especially in the context of infections and cancer [11].

The food industry continuously develops foods and supplements fortified with healthy unsaturated fats, especially polyunsaturated fatty acids such as palmitoleic acid, oleic acid, myristoleic acid, and linoleic acid. These fatty acids are mainly derived from fish oil, however, the increasing pollution of marine environments, including contamination by heavy metals, along with the growing demand for vegetarian and vegan options, has created the need for alternative sources. Microalgae, which are gaining attention as a source of vegetable protein, are recommended as a sustainable alternative food because they are rich in healthy lipids and can be cultured in large quantities [12, 13]. Among microalgae, *Chlorella* is an industrialized, living organism that is mass produced using various established technologies. It has proven safety as a food ingredient, and its efficacy and composition are steadily being studied [14, 15]. While *Chlorella* species typically grow as green cells under light-providing conditions, they are obtained as yellow cells in heterotrophic cultures. This unique coloration arises from the degeneration of chloroplasts in the absence of light during glucose-based cultivation [16, 17].

CV is one of the most extensively studied and commercially utilized *Chlorella* species, particularly in dietary supplements and functional foods, due to its rich content of proteins, polysaccharides, and essential micronutrients [18]. CV also accumulates lipids under certain growth conditions, making it relevant for lipid-based investigations [19]. CP, on the other hand, is notable for its high lipid content, particularly when cultivated in heterotrophic conditions [20]. Given these characteristics, both CV and CP were selected for this study to explore and compare species-specific lipid profiles and their roles in modulating immune responses, thereby providing broader insight into the immune-nutritional potential of *Chlorella*.

Previous research has suggested that various *Chlorella* species can stimulate and regulate the immune system [21-23]. These studies, however, do not provide evidence for how the fractions enriched with lipids can alter the immune system differentially. To our knowledge, there has not been enough research to understand the immune-related benefits of *Chlorella* in conjunction with their lipid metabolome. This study, evaluated the immune-stimulating properties of solvent extracts and fractions of CV and CP. Additionally, lipid profiles were obtained through non-selective LC-MS/MS-based metabolomics analysis. The results of this study might provide insights into the potential use of *Chlorella* as a functional food or dietary supplement for immune system support.

## Materials and Methods

### Chlorella Raw Materials

In this experiment, two microalga types, CV and CP were studied. These microalgae were obtained as green and yellow powders, respectively, from Daesang Corporation (Republic of Korea). The microalgae were dried in their original state and stored in a refrigerator at 4°C, until further use.

### Solvent Extraction, Fractionation, and Isolation

One gram of each of the two samples provided was extracted by refluxing for 1 h using 100 ml of aqueous 70%ethanol, filtered using filter paper, and concentrated under reduced pressure to obtain a crude extract to prepare CV and CP. The crude extract was suspended in distilled water and sequentially fractionated using hexane and butanol to yield CV-Hex, CV-BuOH, CV-Water, CP-Hex, CP-BuOH, and CP-Water. Each was concentrated under reduced pressure to obtain a hexane fraction, a butanol fraction, and a water fraction. Subsequent semi-preparative HPLC of CP-BuOH (MeOH-H_2_O, 30:70, v/v) afforded a single compound with m/z 298.0969 in positive ion mode of high-resolution mass spectrometry (Q Exactive LC-MS, Thermo Fisher Scientific, USA). ^1^H NMR and ^13^C NMR spectra were measured using a 500 MHz spectrometer (Bruker Avance NEO, Germany) equipped with a 5 mm CPP BBO probe. Polysaccharide fractions were prepared using the method of Sui *et al*. [24]. In detail, 10 g of *Chlorella* powder was mixed with 800 ml of distilled water, then 7.5 g of sodium chlorite and 2 ml of glacial acetic acid were added and extracted for 15 h. After centrifugation, the precipitate and supernatant were divided, and the supernatant was immediately mixed with 3x ethanol and centrifuged to obtain soluble polysaccharides (CV-PSA, CP-PSA). The precipitate was recovered by adding hot water (90°C), and the supernatant was centrifuged by adding 3x ethanol to obtain insoluble polysaccharides (CV-PSB, CP-PSB).

### Immune Assay

Total splenocytes were isolated from 6- to 8-week-old C57BL/6 mice spleen. The cells were then cultured using RPMI 1640 medium containing 10% fetal bovine serum, 100 U/ml penicillin G, and 100 μg/ml streptomycin. Then, 3 × 10^5^ cells were seeded in a 24-well plate pre-coated with αCD3/αCD28 and treated. The samples were treated at a concentration of 50 μg/ml. The plates were then maintained in a CO_2_ incubator for 3 days at 37°C with 5% CO_2_. At the end of day 3, the cells were stimulated with PMA/Ionomycin with Golgistop for 4 h for cytokine stimulation before staining. Cytokine production of T cells was confirmed through the expression of TNF-α and IFN-γ.

### Flow Cytometry and Analysis

The cells were washed and labeled with Fixable Viability Dye eF780 (eBioscience, USA) to selectively exclude the dead cells from the analysis. The cells were then surface stained with PE-Cy7-labeled anti-mouse CD4+ or PerCP-Cy5.5-labeled anti-mouse CD8+ anti-body for 30 min in FACS stain buffer, followed by fixation and permeabilization. The cells were then stained for the intracellular cytokines using APC-labeled anti-mouse TNF-α and FITC-labelled anti-mouse IFN-γ for 60 min. The stained cells were then acquired using a FACSVerse flow cytometer (BD Bioscience, USA). Results were then analyzed using Flowjo 10.0 software (Three Star, USA).

### Metabolomics Analysis

LC-MS/MS analysis was carried out using a UHPLC (Vanquish Flex Binary, Thermo Fisher Scientific, USA) connected to a controller, a pump, a degasser, an autosampler, column oven, and a photodiode array detector (Vanquish UHPLC system, Thermo Fisher Scientific) coupled to a mass spectrometer (Q Exactive Quadrupole-Orbitrap, Thermo Fisher Scientific). The analytical column used was a 100 × 2.1 mm, ACQUITY C18 column (Thermo Fisher Scientific). Solvent A consisted of acetonitrile with 0.1% formic acid and solvent B was water with 0.1% formic acid. The chromatography was carried at a 0.3 ml/min flow rate. A linear gradient was applied over 15 min as follows; 0 to 10 min, the mobile phase transitioned from 10% to 100% A; 12.5 to 12.5 min, it was maintained at 100% A; 12.5 to 15 min, the mobile phase returned to 10% A. The injection volume was 5 μl. Full MS spectra were recorded in positive ionization mode covering a mass range of m/z 100 to 1,500 at 70,000 FWHM resolution. MS/MS fragmentation data were obtained in data-dependent mode with 30 V collision energy at 17,500 FWHM resolution. The capillary and cone voltages for the full mass spectrum acquisition were 1.45 kV and 30 V, respectively. The flow rate of helium for the cone gas was 45 L/h, while the flow rate of the desolvation gas (N_2_) was 900 L/h. The desolvation gas temperature was 250°C, while the ion source temperature was maintained at 120°C, and the collision energies for obtaining the MS/MS spectra were set at 15, 20, and 30 V. For the lipidomic analysis, lipid molecules were identified by observation of ions, ensuring a mass accuracy threshold of 5 ppm. Peak integration was carried out using the batch mode of MZmine package (v3.2.8) encompassing various critical steps, such as baseline correction, peak deconvolution, deisotoping, alignment, and gap-filling in accordance with the method described [25]. In addition, peaks with raw intensity values below 1 × 10^4^ were excluded from the analysis.

## Results

### CV-Hex, CV-BuOH, CP-Hex, and CP-BuOH Exhibited Potential Immune-Stimulatory Activity

**Fig. 1:**
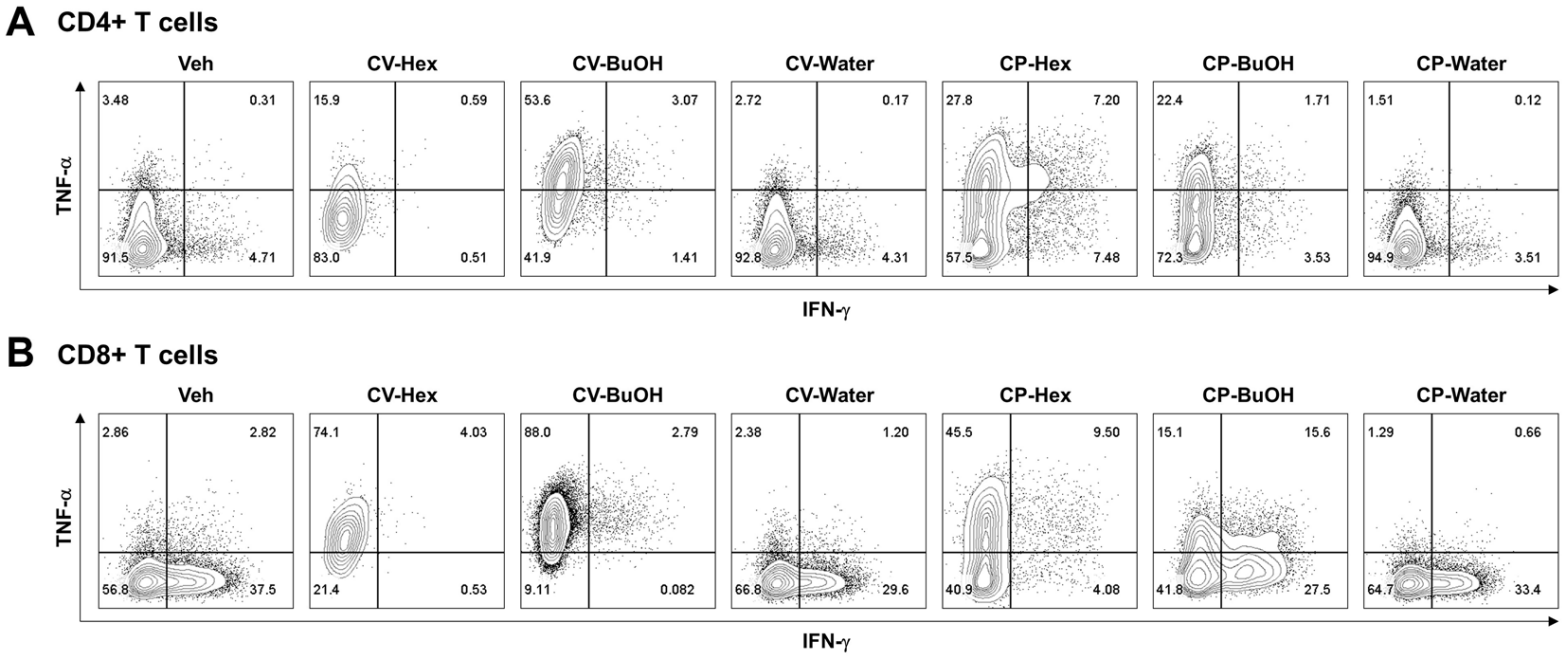
Assessment of cytokine (TNF-α and INF-γ) production profiles of CD4+ T cells and CD8+ T cells in vitro in the presence of different fractions of CV and CP. (**A**) Representative flow cytometry plots showing the levels of TNF-α and INF-γ production gated on CD4+ T cells. (**B**) Representative flow cytometry plots showing the levels of TNF-α and INF-γ production gated on CD8+ T cells.

In this study we investigated the impact of CV and CP on T cell activation by assessing alteration in the effector cytokine production in murine CD4+ and CD8+ T cells. There was a notable trend towards higher TNF-α production in CD4+ T cells treated with the hexane and butanol fractions of both CV and CP (Fig. 1A). In the CP-Hex sample, TNF-α and IFN-γ double producers increased significantly. Similar to CD4+ T cells, CD8+ T cells showed increased TNF-α in the hexane and butanol fractions of both CV and CP (Fig. 1B). Moreover, in the CP-BuOH sample as well, TNF-α and IFN-γ double producers also increased significantly. Overall, the water fractions of CV and CP did not show any differences compared to vehicle control (Veh). These results demonstrated that the Hex and BuOH fractions of CV and CP have immune-stimulating properties.

### Metabolomic Analysis

**Fig. 2:**
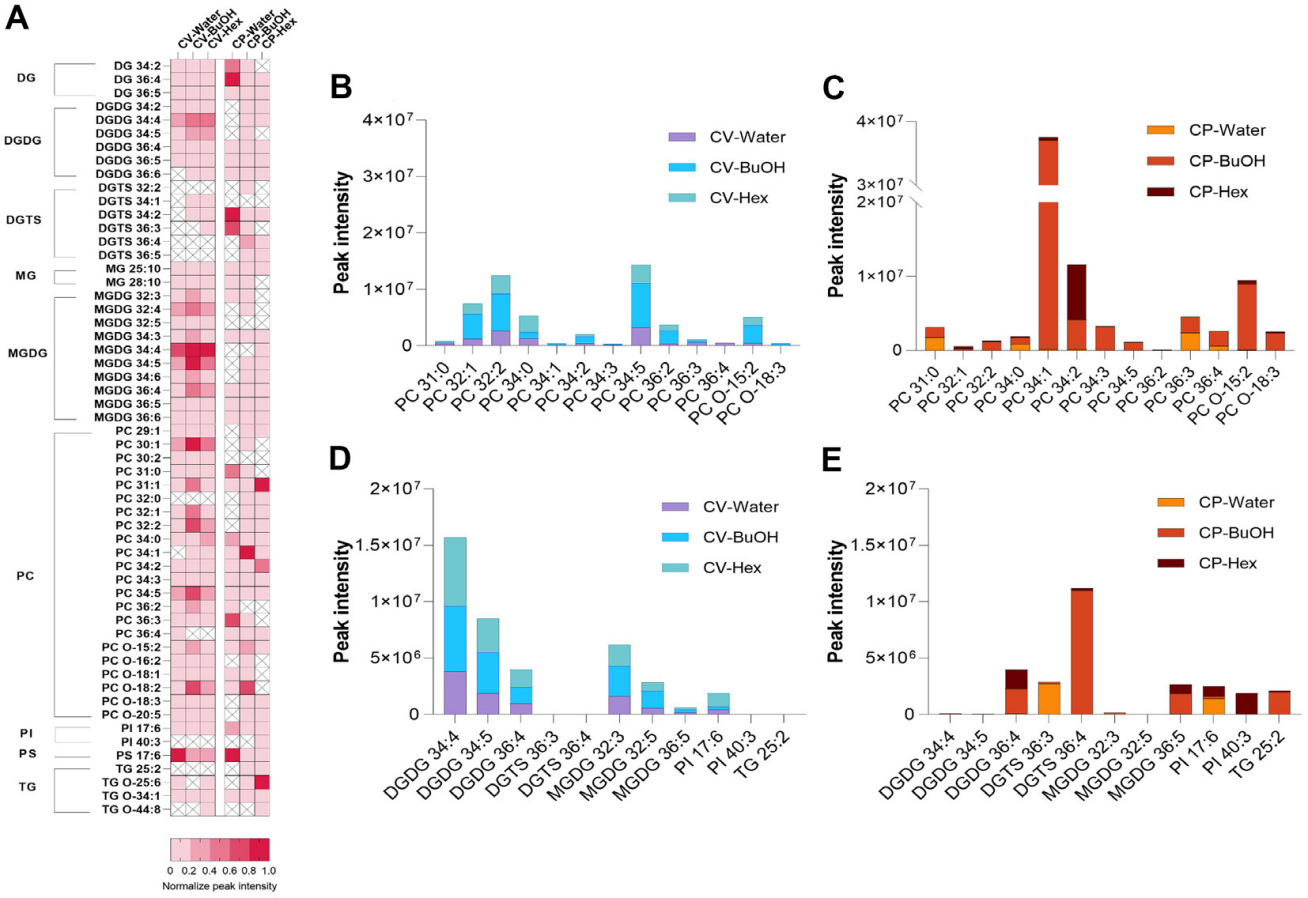
Lipidomic profiling in *Chlorella* solvent fractions detected by LC-MS/MS analysis. (**A**) Heatmap visualization of lipid class identified in each solvent fraction of CV and CP. The assigned values represent the relative proportion of each solvent fraction in different species normalized. Peak intensity of the PC detected in each solvent extract of CV (**B**) and CP (**C**). Peak intensity of the DGDG, DGTS, MGDG, PI, and TG detected in each solvent extract of CV (**D**) and CP (**E**). The clustering of individual lipid species is represented on the left. DG, diacylglyceride; DGDG, digalactosyldiacylglyceride; DGTS, diacylglyceryltrimethylhomoserine; MG, monoglyceride; MGDG, monogalactosyldiacylglyceride; PC, phosphatidylcholine; PI, phosphatidylinositol; PS, phosphatidylinositol; TG, triacylglyceride.

*Chlorella* is typically rich in lipids and polysaccharides [15]. The immunological effects of polysaccharides have been reported [26, 27], so we prepared polysaccharide fractions from CV and CP and evaluated their effects on T cells. Although we did not see a similar pattern of TNF-α increase as shown in Fig. 1, an overall mild induction of IFN-γ was exhibited (Fig. S1). In addition, carotenoids, especially lutein, are also found in high amounts in *Chlorella*. Unlike CP, which was harvested from heterotrophic culture dark conditions, lutein was identified in extracts and fractions from CV (Fig. S2) [28]. CV and CP showed immunomodulatory activities, and thus we became interested in *Chlorella* lipids. Then, the solvent fractions of CV and CP obtained using hexane, butanol, and water were analyzed by LC-MS/MS to determine their lipid profiles. This allowed us to identify a variety of polar lipids, including phospholipids and glycolipids, as well as neutral lipids, such as triglycerides. By using lipidomic methods, we identified 50 and 53 lipid species above the intensity threshold of 1x10^4^ from all CV and CP samples, respectively. These classes include sulfoquinovosyldiacylglycerol (SQDG), digalactosyldiacylglycerol (DGDG), digalactosylmonoacylglycerol (DGMG), monogalactosyldiacylglycerol (MGDG), monogalactosyl-monoacylglycerol (MGMG), phosphatidylcholine (PC), phosphatidylethanolamine (PE), phosphatidylglycerol (PG), phosphatidylinositol (PI), lysophosphatidylcholine (LPC), lysophosphatidylethanolamine (LPE), ceramide (Cer), and inositolphosphoceramide (PI_Cer). The detected polar lipid species and their detection intensities are summarized in Table 1 and Fig. 2. Among the detected lipids, PC 32:1, PC 32:2, PC 34:5, DGDG 34:4, DGDG 34:5, and MGDG 32:3 were abundant in the butanol and hexane fractions of CV, while PC 34:1, PC 34:2, PC O-15:2, and DGTS 36:4 were enriched in the butanol and hexane fractions of CP. For CV, the lipid composition of the hexane and butanol fractions was not significantly different, but for CP it was, and this may explain the differential increase in IFN-γ in CD4+ and CD8+ T cells by treatment of the CP fractions.

**Fig. 3:**
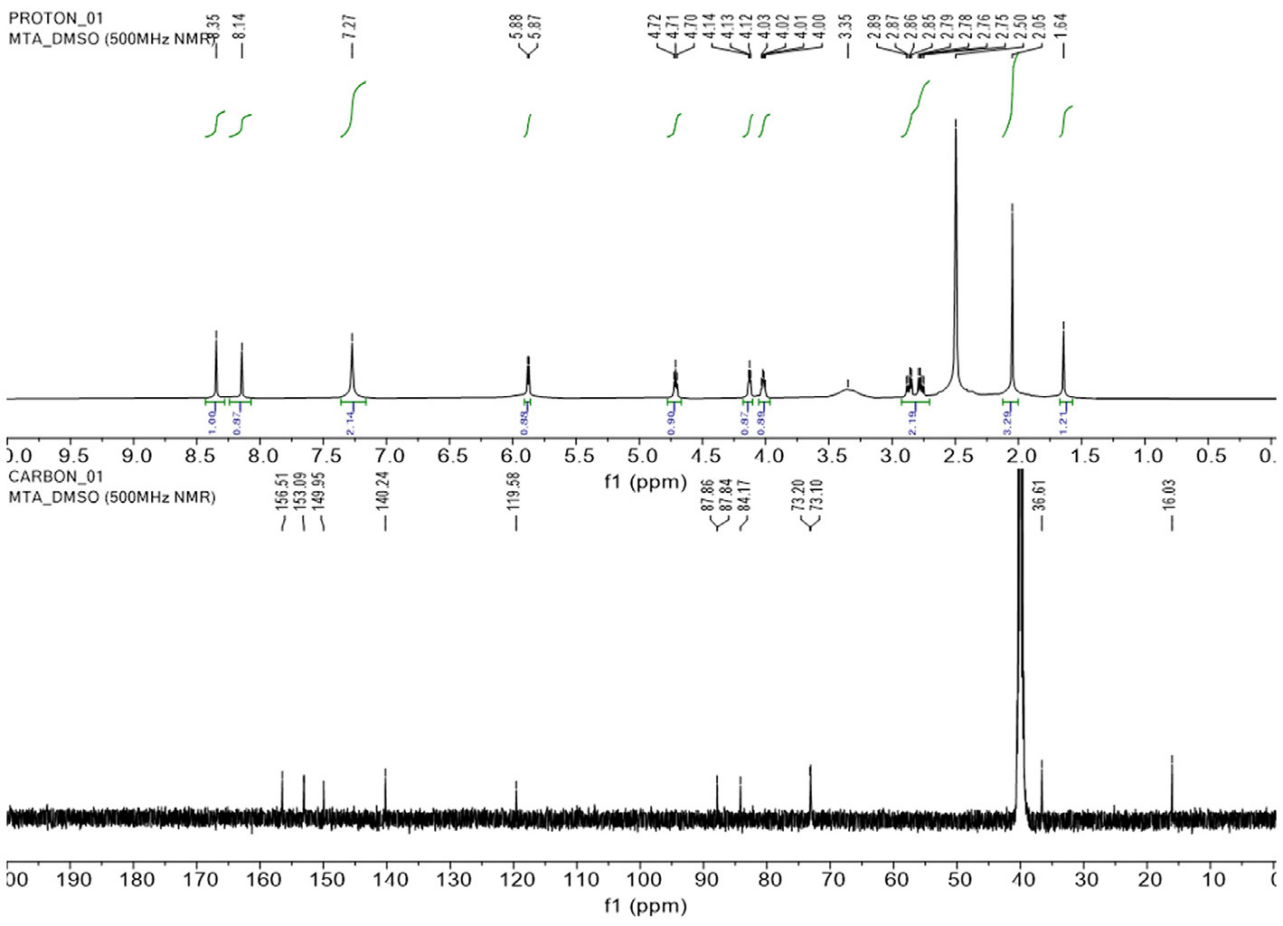
1H (upper) and ^13^C NMR (bottom) spectra of 5’-methylthioadenosine isolated from CV.

During LC-MS analysis of the *Chlorella* ethanol extracts (CV, CP), a peak with a molecular ion of 298, common to both extracts, was detected and identified as 5'-methylthioadenosine (MTA) by UV spectrum and high-resolution mass spectrometry (Fig. S3). The structure of MTA was confirmed by NMR analysis (Fig. 3) after separation using preparative HPLC [29]. In detail, the ^1^H NMR spectrum showed the representative proton resonances for adenosine and an additional singlet methyl signal at 2.05 ppm for S-CH_3_ to construct MTA, evidenced by the ^13^C NMR spectrum indicating the presence of eleven carbons. 5’-methylthioadenosine: colorless liquid, m/z 298.0969 [M+H]^+^ (calcd for C_11_H_16_N_5_O_3_S, 298.0974). Fig. 3 presents the ^1^H NMR spectrum (500 MHz, DMSO-*d*_6_) and ^13^C NMR spectrum (125 MHz, DMSO-d6).

### Immunostimulatory Effect of MTA

**Fig. 4:**
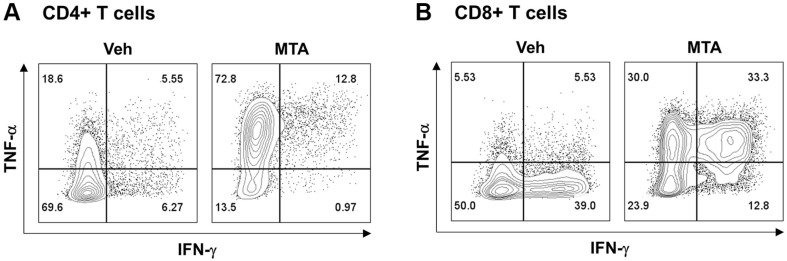
Evaluation of TNF-α and INF-γ production in CD4+ and CD8+ T cells in vitro in the presence of 50 μg/ml of MTA. (**A**) Representative flow cytometry plots showing the levels of TNF-α and INF-γ gated on CD4+ T cells. (**B**) Representative flow cytometry plots showing the levels of TNF-α and INF-γ gated on CD8+ T cells.

MTA is a naturally occurring nucleoside that has gained significant interest in scientific research due to its potential therapeutic benefits, including regulation of the immune response by modulating the activity of T cells, B cells, and natural killer cells [30, 31]. We therefore tested the immune-stimulating activity of MTA in T cells (Fig. 4). In CD4+ T cells, MTA can induce significant TNF-α production accompanied with the increase in the TNF-α and IFN-γ double producers compared to the vehicle control (Fig. 4A). Like the above, TNF-α only and double producers were increased in MTA-treated CD8+ T cells (Fig. 4B).

## Discussion

Earlier investigations exploring the immunomodulatory potential of *Chlorella* have primarily focused on hot-water extracts or water-soluble polysaccharide fractions, which have consistently been shown to enhance various immune parameters. For instance, a previous study reported that CV hot-water extract enhanced the production of IL-2, IL-12, TNF-α, and IFN-γ in *Listeria monocytogenes*-infected mice [32]. Similarly, An *et al*. demonstrated increased IFN-γ and IL-2 production in the human T-cell line MOLT-4 following CV water extract treatment [33]. Clinical studies further support these findings, with *Chlorella* supplementation enhancing salivary secretory IgA levels and NK cell activity [21], pointing to the immunostimulatory nature of its polysaccharide-rich components.

Supporting this line of evidence, another study showed that the hot-water extract and polysaccharides extracted from CP augmented IL-1β and TNF-α expression and enhanced phagocytic activity in macrophages via the TLR4 signaling pathway [34]. Additional work confirmed upregulation of IL-1β and TNF-α mRNA following CP polysaccharide fraction treatment [35], further reinforcing the role of polysaccharides in immune activation.

In contrast to these polysaccharide-centric approaches, the present study utilizes lipid-enriched solvent fractions, specifically the hexane and butanol extracts of CV and CP as the primary contributors of immunostimulatory activity. These fractions significantly increased TNF-α and IFN-γ production in both CD4+ and CD8+ T cells. This increased cytokine production indicates a robust pro-inflammatory and immune activating potential [36]. In our study, the water fractions showed only mild immune effects, likely linked to residual polysaccharides, whereas the lipid fractions elicited robust cytokine response.

TNF-α and IFN-γ produced by CD4+ and CD8+ T cells are key mediators of immune defense, inflammation, and immune regulation. In CD4+ T cells, IFN-γ activates macrophages and promotes a Th1 response critical for the clearance of intracellular pathogens like *Leishmania*, while TNF-α supports this by inducing metabolic reprogramming through ITK-Akt-mTOR pathway, facilitating differentiation into proinflammatory T cell subsets [36, 37]. In CD8+ T cells, TNF-α is rapidly produced upon activation and plays a role in modulating dendritic cell activity, shaping early immune responses [38]. Although essential for effective immune function, excessive production of these cytokines has been associated with immunopathology, including rheumatoid arthritis and chronic infections [39]. The synergistic action of TNF-α and IFN-γ enhances antimicrobial activity and T cell fitness, highlighting their therapeutic relevance and the importance of maintaining a balanced immune response.

To better understand the active components within these fractions, lipidomic profiling was conducted. Lipidomic analysis by non-targeted LC-MS/MS method identified a total of 56 molecular ions corresponding to *Chlorella* lipids, 50 from CV and 53 from CP. These ions were categorized into nine lipid classes: DG, DGDG, DGTS, MG, MGDG, PC, PI, PS, and TG. While the types of lipids in CV and CP were generally similar, some lipid species were specific to each *Chlorella* species. For example, DGTS 32:2, DGTS 36:4, GDGS 36:5, PC 32:0, and PI 40:3 were found only in CP, while DGTS 34:1, MGDG 32:5, and PC 30:2 were detected exclusively in CV. These species-specific lipids might be instrumental in *Chlorella* identification.

This work also noted variations in the peak intensities of certain lipid species between CV and CP, depending on the solvent used for fractionation. For example, PC 34:1 showed high intensities in CP-BuOH, but negligible amounts in CV. Phospholipids like phosphatidylcholine (PC) are vital structural components of cell membranes, contributing to cell integrity and function. Interestingly, PC intensity levels varied depending on the extraction solvent, with higher levels observed in the butanol and hexane fractions. The choice of solvent for lipid extraction is critical and should be based on the lipid profile of each solvent to optimize extraction efficiency.

Several lipid classes identified in our active fractions may contribute to the observed immunostimulatory effects. Phospholipids, such as PC and PE, are major structural components of cellular membranes in *Chlorella*. LPC has been reported to have immunomodulatory functions, including the promotion of T cell activation and the recruitment of immune cells like monocytes [40]. Glycolipids have also been shown to activate macrophages and enhance phagocytic activity, with studies demonstrating a dose- dependent increase in the expression of Il-1β, Il-6, and TNF-α [41]. Supporting these observations, a study on *Chlorella sorokiniana* found that its cell wall extract significantly increased mRNA levels of TNF-α and IFN-γ in human lymphoblasts [42], which align with the cytokine profile observed in our study. Along with lipidomic analysis, we confirmed the immunostimulatory effect of MTA, a common component in both active fractions of CV and CP. MTA, a natural sulfur-containing biomolecule produced during the metabolism of S-adenosylmethione, has been the subject of research due to its potential biological activities, including suppression of tumors, neuroprotection, and immunomodulatory activity [30, 43, 44]. Moreover, its lack of toxicity concerns, even at high doses [45, 46], might be an advantage that promotes research and development on *Chlorella* as an immune-enhancing food material. Lipidomic analysis revealed species-specific differences in lipid compositions, with PC 32:1, PC 32:2, PC 34:5, DGDG 34:4, and DGDG 34:5 being enriched in CV active fractions, while PC 34:1, PC 34:2, PC O-15:2, and DGTS 34:4 were abundant in CP fractions. Additionally, we identified 5'-MTA as a common bioactive component in both *Chlorella* species with immunostimulatory properties. These findings provide a scientific foundation for developing *Chlorella*-based functional foods and dietary supplements aimed at immune system support, with potential applications tailored to specific desired immune responses.

Despite these promising findings, the study has certain limitations, notably the in vitro nature of the immune assays. To fully validate the immunostimulatory potential of MTA and the lipid fractions under physiological conditions, comprehensive in vivo studies are required. Additionally, investigations into receptor-mediated uptake and the associated intracellular signaling pathways would provide deeper mechanistic insights into how these bioactive compounds exert their effects.

In summary, this study highlights the immune-stimulating potential of CV and CP lipid fractions, particularly those extracted with hexane and butanol. Unlike previous reports that primarily focused on water-soluble polysaccharides, our findings reveal that lipid-rich fractions can also robustly activate CD4+ and CD8+ T cells, promoting the production of TNF-α and IFN-γ. Furthermore, the identification of MTA as a shared immuno-stimulatory metabolite in both species further underscores the presence of potent, bioactive lipid-associated molecules. These results not only expand the current understanding of the bioactivity of *Chlorella* but also suggest a shift in focus from immune activation by *Chlorella* polysaccharides to immune activation through lipid components. The observed lipidomic differences between species highlight the importance of developing species-specific formulations of *Chlorella*-based supplements. Taken together, this work provides a compelling scientific rationale for the development of lipid-enriched, *Chlorella*-based functional foods, tailored to enhance specific immune responses, with promising applications against infection and cancer treatment.

## Supplemental Materials

Supplementary data for this paper are available on-line only at http://jmb.or.kr.



## Figures and Tables

**Table 1:** List of lipids in *Chlorella* solvent fractions detected by LC-MS/MS analysis.