# Supplementary material for: Chemical Profiling and Immune-Stimulating Activity of Solvent Fractions Derived from Dietary Chlorella
Source: J Microbiol Biotechnol. 2025 Jun 12;35:e2503021. doi: 10.4014/jmb.2503.03021 (PMC12197813; doi:10.4014/jmb.2503.03021)
Supplement: Supplementary file 1 [file jmb-35-e2503021-supple.pdf]

Figure S1

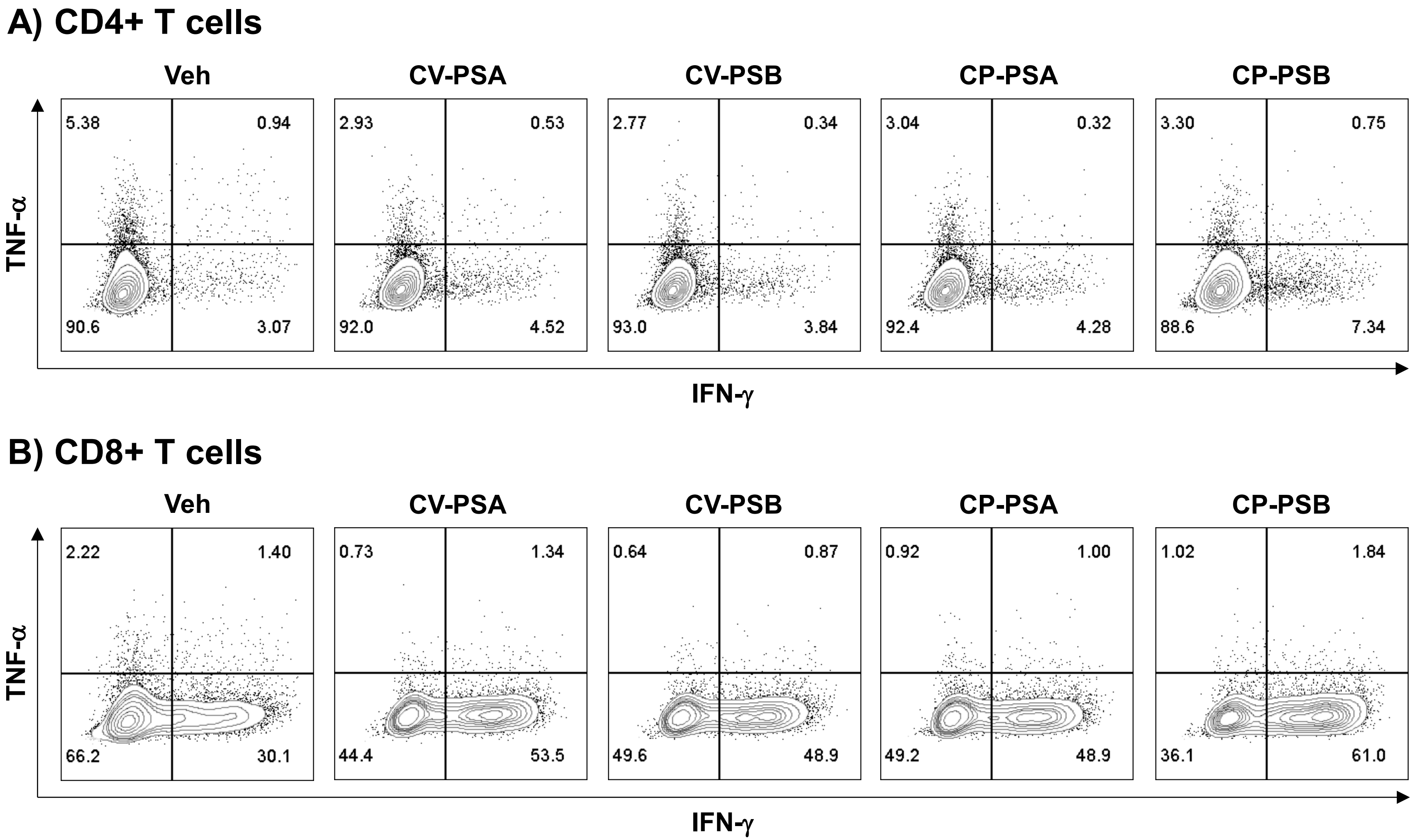

Figure S1. Effects of Chlorella polysaccharide fractions on TNF-α and IFN-ɣ production in murine CD4+ and CD8+ T cells.

Figure S2

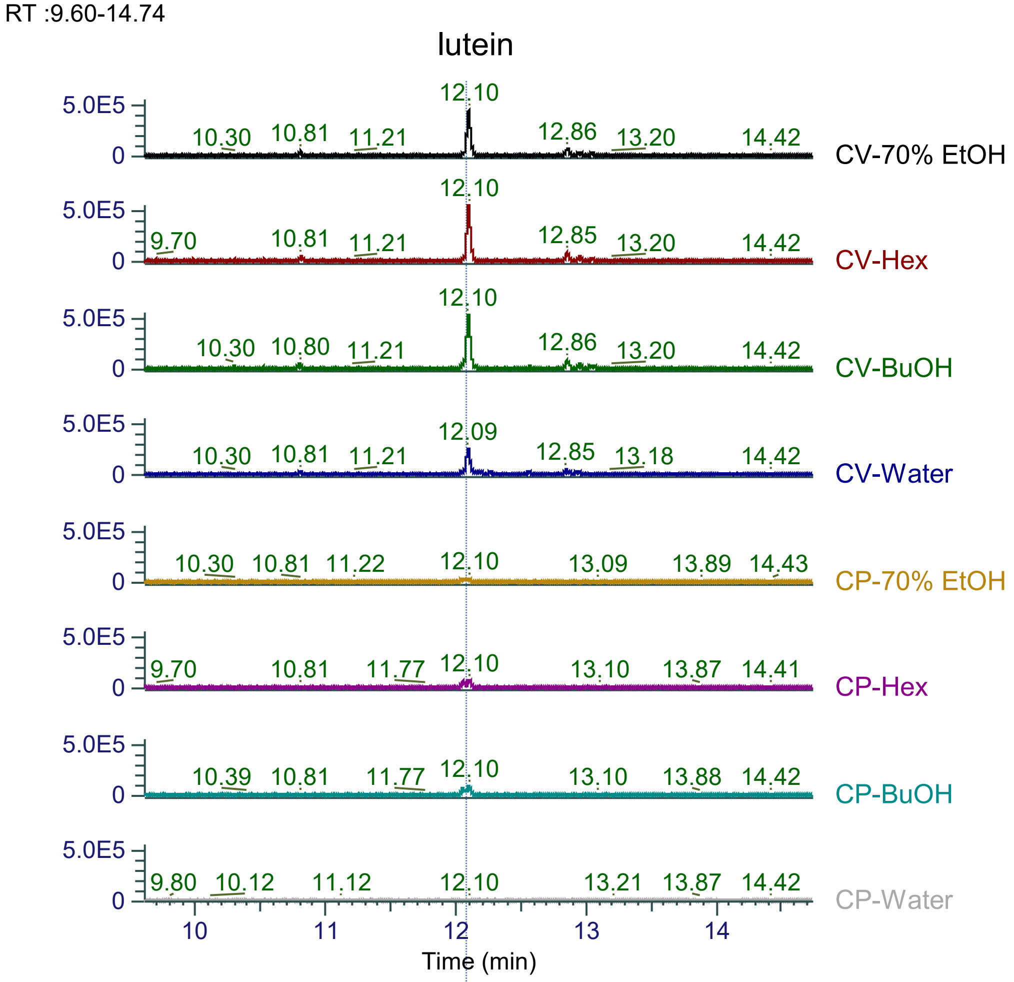

LC chromatograph indicating lutein

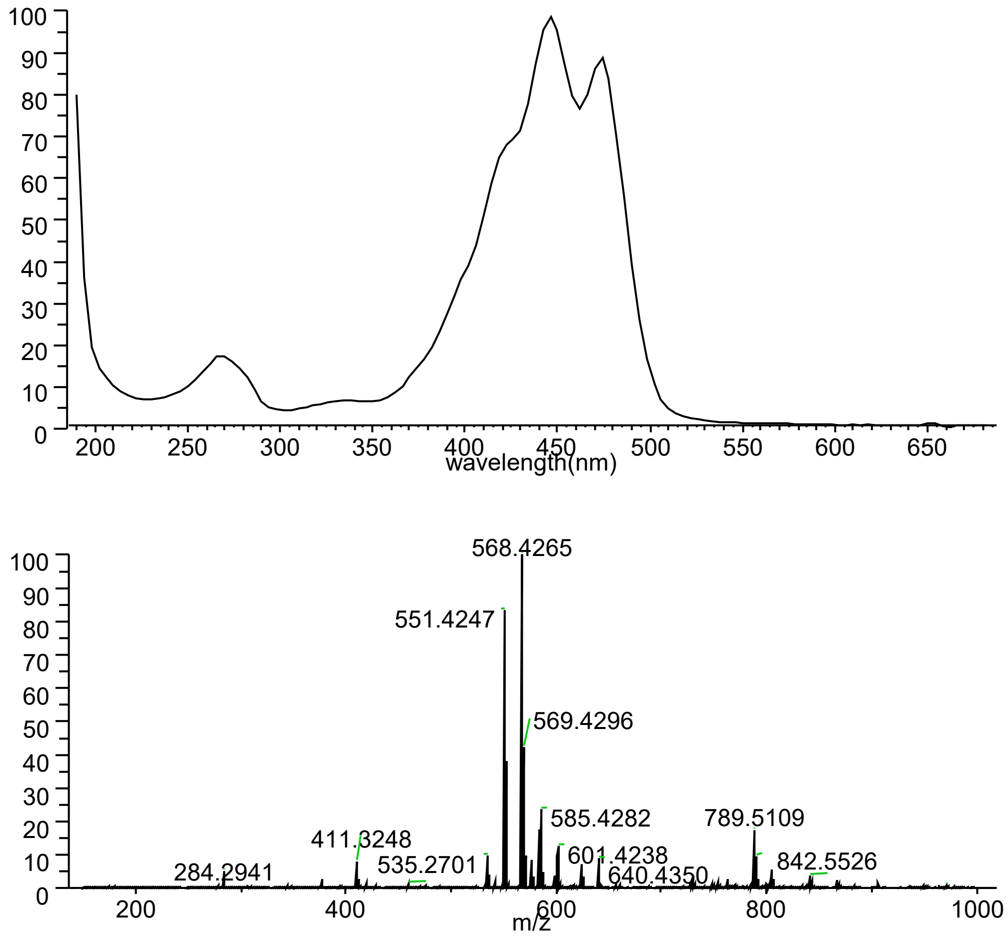

UV and mass spectrum of lutein found in chlorella

Figure S2. LC-MS data including UV spectrum and mass spectrum of lutein from extracts and fractions. 568.4265 for [M·]+, 551.4247 for [M-H_2O+H]+

Figure S3

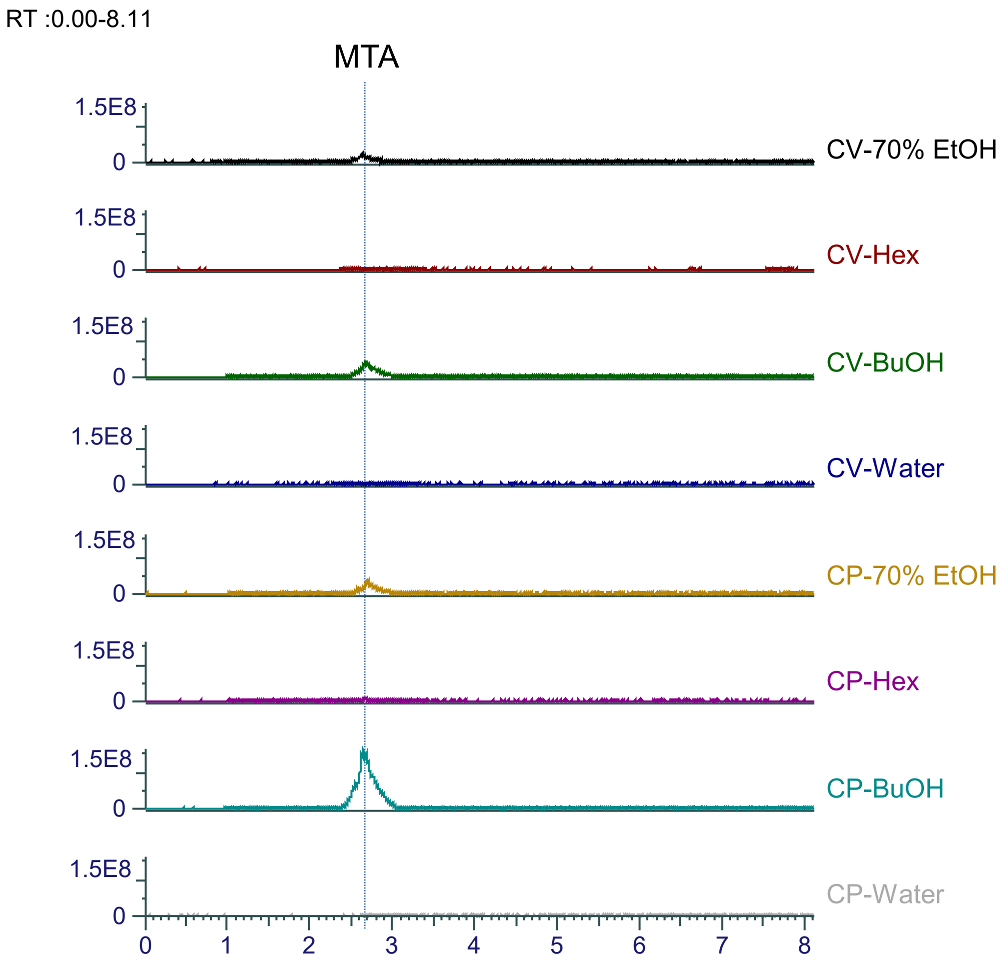

LC chromatograph indicating MTA

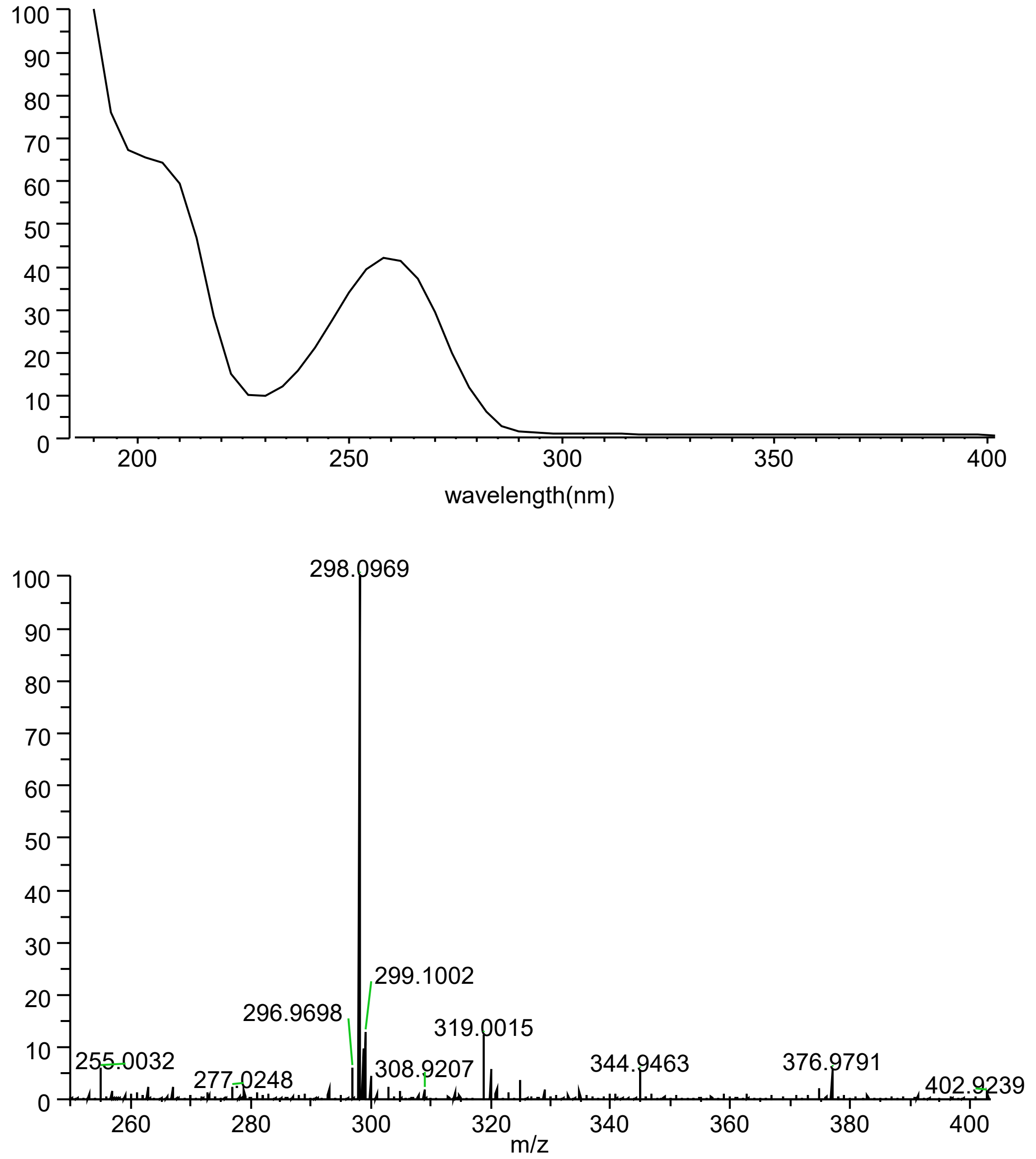

UV and mass spectrum of MTA found in chlorella

Figure S3. LC-MS data including UV spectrum and mass spectrum of 5'-methylthioadenosine (MTA) from extracts and fractions.
